# Clinical Profile of Patients with Rare Inherited Coagulation Disorders: A Retrospective Analysis of 67 Patients from Northern India

**DOI:** 10.4084/MJHID.2012.057

**Published:** 2012-10-02

**Authors:** Sanjeev Kumar Sharma, Suman Kumar, Tulika Seth, Pravas Mishra, Narendra Agrawal, Gurmeet Singh, Avinash Kumar Singh, Manoranjan Mahapatra, Seema Tyagi, Haraprasad Pati, Renu Saxena

**Affiliations:** Department of Hematology, All India Institute of Medical Sciences, New Delhi, India

## Abstract

**Introduction:**

Inherited bleeding disorders are characterized by the absence or reduced level of clotting factors, and the clinical manifestations vary according to the type and magnitude of the deficient factor.

**Aim:**

To study the clinical presentation of the rare inherited coagulation factor disorders in a tertiary care hospital and to compare the data from those reported in other populations.

**Methods:**

Sixty-seven patients, who presented to the Department of Hematology, All India Institute of Medical Sciences, New Delhi, were evaluated retrospectively from 2005 to 2011. The tests performed included platelet count, prothrombin time (PT), activated partial thromboplastin time (aPTT), thrombin time (TT), factors assay and clot solubility test in 5 M urea. Factor XI assays were aPTT based while factors V, VII and X assays were PT based.

**Results:**

Male to female ratio was 2:1. The median age of onset of the first episode of bleeding was at 6 months (range, from birth to 20 years) whereas the median age of presentation to our hospital was 9 years (range, 2 months to 54 years). The most common deficient factor was factor X (43%), followed by factor XIII (27%) and factor VII (10%).

**Conclusion:**

There is a wide gap between the initial manifestation of the bleeding disorders and first presentation to the tertiary care hospital for assessment and treatment. Factor X deficiency is the most common among these rare coagulation disorders in our population, whereas factor VII deficiency is more common in Iranian and North American population.

## Introduction

Inherited bleeding disorders are characterized by the absence or reduced levels of clotting factors, and the manifestations vary according to the type and magnitude of the deficient factor. Common coagulation disorders include those caused by the deficiencies of factors (F) VIII, IX, von-Willebrand factor and platelet function defects. Rare inherited factor disorders include deficiencies of factors II, V, VII, X, XI, XIII and fibrinogen. Though these factor deficiencies are rare in the general population, they may lead to serious hemorrhagic manifestations and even death, if not diagnosed and treated. We studied the clinical presentation and manifestation of these rare inherited coagulation disorders in a tertiary care hospital and compared the data from those reported in other populations. Increased awareness is needed for early referral and management.

## Study Material

### Subjects

Sixty-seven patients, who presented to the Department of Hematology, All India Institute of Medical Sciences, New Delhi, were evaluated retrospectively from 2005 to 2011. A detailed history of drug intake, consanguinity, and family history of bleeding disorders was recorded.

### Coagulation tests

The tests performed included platelet count, prothrombin time (PT), activated partial thromboplastin time (aPTT), thrombin time (TT), factors assay and clot solubility test in 5 M urea. PT and aPTT were measured by using the kit (Diagnostica Stago). The normal values of PT and aPTT were 11–15 secs and 29–35 secs, respectively. Different coagulation factors assays were performed on the basis of initial testing. Factor XI assays were aPTT based while factors V, VII and X assays were PT based. Deficient plasma and other reagents for factor assay were taken from Diagnostics Stago. Reference values for all aPTT based factor activity were taken between 60% and 150% and those for PT based factor activity were taken between 70% and 150% that have been set by the kit provider. On the basis of the plasma levels of FX coagulant activity measured with a PT based assay using thromboplastin and FX-deficient plasma, patients were classified into three groups i.e. severe (<1%), moderate (1%–5%) and mild (6%–10%). Clot solubility test, using a solution of 5 molar urea, was used for the diagnosis of F XIII deficiency.

## Results

A total of 67 patients were studied (Table 1). Male to female ratio was 2:1. The median age of onset of the first episode of bleeding was at 6 months (range, from birth to 20 years) whereas the median age of presentation to our hospital was 9 years (range, 2 months to 54 years). The most common deficient factor was factor X (43%), followed by factor XIII (27%) and factor VII (10%). Four patients had Factor V deficiency. Factor XI deficiency and combined factor V and VIII deficiency was seen in one patient each. Mucocutaneous bleeding was the most common presenting complaint seen in 65% of patients. History of umbilical stump bleeding was present in 18% of patients, 67% of whom had factor XIII deficiency. A total of 13 patients (20%) developed intracranial hemorrhage (ICH). Factor XIII deficiency was the most common risk factor for ICH, with 50% of patients with ICH having factor XIII deficiency. Three patients presented with hemarthrosis while 14 patients developed hemarthrosis during follow-up. Knee joint was most commonly involved (12 out of 17 patients). History of consanguity was present in 15% of cases. Significant family history of bleeding was present in 19% of patients with maximum incidence in patients with factor XIII deficiency (39%). Most of the patients were treated with antifibrinolytics and fresh frozen plasma.

### Factor X deficiency

It was the most common deficiency disorder among all of the rare factor deficiency disorders, accounting for about 43% of total cases. Twenty-one patients were male and 8 were females. History of consanguity was present in 7% of cases. Patients were classified based on the FX coagulation activity. Twenty-two patients (76%) had severe FX deficiency (FX levels <1%), 7% had moderate (1–5%) and 17% had mild deficiency (6–10%). Mucocutaneous bleeding was the most common presentation with ecchymosis occuring in 55% followed by epistaxis or gum bleeding in 27% patients. Seven patients with severe FX deficiency (24%) had hemarthrosis and two developed pseudotumor. Four patients (13%) had intracranial hemorrhage, three of whom were neonates with severe factor deficiency, who succumbed to the bleed. Two of the three women in the reproductive age group had menorrhagia. One patient with severe deficiency of FX developed pericardial tamponade which required emergency drainage with fresh frozen plasma (FFP) support. Most of the patients were managed with antifibrinolytics (tranexamic acid 10 mg/kg/dose 3–4 times/day) and FFP transfusion (10–15ml/kg).

### Factor XIII deficiency

Factor XIII deficiency was the second most common inherited coagulation factor disorder (27%). Diagnosis was made based on the 5-molar urea clot-solubility test. This clot solubility test is only sensitive at very low levels of FXIII (<1%). There were 11 males and 7 females. History of consanguity was present in 33% of cases. Eight out of the 18 patients (44%) had a history of umbilical stump bleeding. Eleven patients had mucocutaneous bleed and 2 patients had prolonged bleeding following minor trauma. Intracranial hemorrhage occurred in 8 (44%) patients (7 intracerebral and 1 subdural) and hemarthrosis in 3 patients. Two patients developed pelvic hematomas and 1 patient developed a thigh hematoma. There were 3 females in the reproductive age group, one of whom one had recurrent miscarriages (8 times). Patients were managed with antifibrinolytics and monthly FFP transfusion, though they had poor compliance towards monthly prophylactic FFP transfusions. Only six out of the 18 patients with FXIII deficiency followed regular FFP prophylaxis. One patient who left the prophylactic monthly FFP transfusions presented with intracranial hemorrhage 6 months later and died.

### Factor VII deficiency

Factor VII deficiency was reported in 7 patients (5 males and 2 females). The severity of Factor VII deficiency in these patients was graded as per the clinical presentation.^1^ Three patients had hemarthrosis, 2 of whom had factor VII levels less than 1% and one patient had FVII levels of 12%. These patients were considered to have clinically severe bleeding disorder. Two patients had only mucocutaneous bleedings, with factor levels < 1% and 3% respectively. The other 2 patients were diagnosed when they presented with prolonged post-traumatic bleeding, both of these patients had factor VII levels less than 1%.

One 35 years old male patient with factor VII levels less than 1% underwent successful cholecystectomy for cholelithiasis under cover of recombinant FVIIa. He had mild mucocutaneous bleeds previously. He was given 20μg/kg recombinant FVIIa twice daily for 5 days from the day of surgery.

### Fibrinogen deficiency

A-or hypofibrinogenemia was diagnosed in five patients. History of consanguity was present in 33% of cases. Family history of bleeding was present in two patients. One patient had severe fibrinogen deficiency (<10mg/dl), and four had moderate deficiency with fibrinogen levels between 10–30mg/dl. Umbilical stump bleeding (43%) and hemarthrosis (43%) were the most common manifestations in these patients. Two patients had dysfibrinogenemia. One patient with dysfibrinogenemia presented at the age of 54 years with spontaneous hemarthrosis. The other was a one year old girl who presented with recurrent gum bleedings. She was found to have autosomal dominant congenital dysfibrinogenemia. She was started on prophylactic fibrinogen concentrate which prevented further bleeding.

### Factor V deficiency

There were four patients with factor V deficiency. Three patients had severe deficiency (factor V activity levels <1%). One of these patients presented with ecchymosis but later developed ICH, one presented with muscle hematoma and one developed knee hemarthrosis. The fourth patient with moderate deficiency (factor V activity 2.5%) presented with ecchymoses. Factor VIII coagulant activity was also measured in all patients to exclude the combined deficiency of factors V and VIII.

### Other rare bleeding disorders

One child was diagnosed with factor XI deficiency. He presented with recurrent epistaxis at the age of 15 years. Another child had a combined deficiency of factor V (20%) and VIII (6%). He presented with ecchymosis at the age of 6 years, without any major bleeding episodes.

## Discussion

Patients with inherited deficiencies of clotting factors have a lifelong risk of bleeding which can range from minor to catastrophic hemorrhages. The bleeding pattern has earlier been reported from India,^2^–^4^ but this study investigated a larger number of patients from Northern India, with emphasis on the clinical manifestations and pattern of bleeding in the rare inherited coagulation disorders. We also compared our data with those from other populations (Table 2). Muco-cutaneous bleeding was the most common presenting complaint seen in the majority of patients (65%). In Iranian, Italian and North American populations, factor VII deficiency is the most common rare inherited coagulation disorder,^5^–^7^ in contrast to our population which has FX deficiency as the most common rare bleeding disorder.^2^–^4^ There is also regional variation in the rare inherited coagulation disorders within India with FXIII deficiency being most common in Southern India.^8^

Factor X deficiency is considered as the most severe recessively inherited rare bleeding disorder.^9^ In the Iranian study, epistaxis occurred in 72% of patients, followed by hemarthrosis in 69%, umbilical stump bleeding in 28% and CNS bleed in 9%.^9^ Similarly, in the European and Latin American study, spontaneous bleeding in F X deficient patients comprised of easy bruising (55%), hematoma (43%), epistaxis (36%), hemarthrosis (33%), intracranial hemorrhage (21%), and gastrointestinal (GI) hemorrhage (12%).^10^ In our patients with F X deficiency mucocutaneous bleeds (82%) were more common, with ecchymosis occuring more frequently than epistaxis. Incidence of hemarthrosis (13%) was lower than that reported in the Iranian population but similar to that reported in the North American registry.^6^,^9^ Intracranial hemorrhage is a significant risk for early mortality,^11^ as noted in our study.

In patients with F XIII deficiency, ecchymosis was the most common symptom as has been reported earlier also.^12^ In the Iranian population with factor XIII deficiency, umbilical stump bleeding occurred in 73% patients, 55% patients had hemarthrosis and 25% patients had CNS bleeding.^13^ The incidence of intracranial haemorrhage has been reported to be 25–30% but was higher (44%) in our series. In patients with factor VII deficiency, there has been a weaker correlation of severity of bleeding with factor levels.^1^,^14^ Joints were the primary site of bleeding in our patients whereas in Iranian and Italian patients, epistaxis and menorrhagia were the presenting complaints.^5^,^15^ In patients with severe factor VII deficiency, bleeding into the central nervous system is common and has been reported between 15% and 60% of cases,^5^,^16^ though none of our patients had CNS hemorrhage.

Among the patients with severe fibrinogen deficiency (< 10mg/dl) reported by Peyvandi et al,^17^ the most frequent hemorrhage was hemarthrosis (25%). We also observed hemarthrosis frequently in our patients (43%). Though the majority of patients with dysfibrinogenemia do not bleed^18^ and rather present with thrombosis,^19^ our patients had recurrent gum bleeds and epistaxis. Though there were few patients with factor V deficiency in our series, mucocutaneous, musculoskeletal and intracranial bleeds correlated with activity levels with similar pattern as reported in Iranian and North American patients.^17^,^20^ Patients with factor XI deficiency can have variable bleeding, both mucocutaneous and musculoskeletal bleeds can occur, and are not affected by severity of factor deficiency.^21^ Our patient had only epistaxis. Patients with combined factor V and factor VIII usually have milder symptoms,^22^,^23^ our patient also had ecchymosis only.

Most of our patients (> 80%) were managed with FFP transfusions (10–15ml/kg) on demand. Oral antifibriniolytics (tranexamic acid 10 mg/kg/dose 3–4 times/day) were the most commonly used drugs for minor bleeding episodes.

## Conclusion

Though first episode of bleeding occurs early in the life, most patients are not diagnosed till later in childhood or even adulthood. There is a wide gap between the initial manifestation of the bleeding disorder and first presentation to the tertiary care hospital for assessment and treatment. Factor X deficiency was the most common among these rare coagulation disorders. Factor XIII deficiency was the second most common inherited disorder found in our population and was the one in which intracranial hemorrage was a major risk factor. The incidence of these rare coagulation disorders is underdocumented and may be more common than what is reported.

## Figures and Tables

**Table 1:** Patient features with rare inherited coagulation factor disorders.

Factor deficiency	Number of patients (%)	Male: Female	Median age of first bleed (yrs)	Median age of presentation to tertiary care centre (yrs)
F I	7 (10%)	3:4	0.09	8.5
F V	4 (6%)	4:0	0.87	5.5
F V+VIII	1 (1.5%)	0:1	6	9
F VII	7 (10%)	5:2	1	15
F X	29 (43%)	21:8	0.58	6
F XI	1 (1.5%)	0:1	15	25
F XIII	18 (27%)	11:7	0.29	8
Total	67	2:1	0.5	9

**Table 2:** Clinical profile of our patients with rare inherited coagulation disorders compared with Iranian and North American population.

Factor deficiency	Percentage of patients			Musculoskeletal (Hemarthrosis/muscle hematoma)			Muco-cutaneous bleed			CNS bleed			Umbilical cord bleed		
	Iran n=750	North America n=285	Present study n=67	Iran	North America	Present study	Iran	North America	Present study	Iran	North America	Present study	Iran	North America	Present study
F I	11%	10%	10.44%	54%/72%	26%	42.8%	72%	49%	28.5%	10%	5%	0	85%	-	57.1%
F V	7%	12.9%	5.97%	26%/29%	23%	50%	57%	53%	75%	6%	8%	25%	3%	-	0
F VII	39%	46%	10.44%	20%/10%	18%	57%	65%	70%	28.6%	15%	3%	0	0	-	0
F X	10%	11.9%	43.28%	69%/66%	27%	27.4%	72%	45%	82%	9%	15%	13%	28%	-	3.4%
F XIII	13%	8.3%	26.86%	55%/58%	27%	33%	80%	25%	61.1%	25%	10%	44%	73%	22%	44%

## References

[1] Lapecorella M, Mariani G (2008). Factor VII deficiency: defining the clinical picture and optimizing therapeutic options. Haemophilia.

[2] Kashyap R, Saxena R, Choudhry VP (1996). Rare inherited coagulation disorders in India. Haematologia.

[3] Gupta M, Bhattacharyya M, Choudhry VP, Saxena R (2005). Spectrum of inherited bleeding disorders in Indians. Clin Appl Thromb Hemost.

[4] Ahmad F, Kannan M, Ranjan R, Bajaj J, Choudhary VP, Saxena R (2008). Inherited platelet function disorders versus other inherited bleeding disorders: An Indian overview. Thrombo Res.

[5] Peyvandi F, Mannucci PM, Asti D, Abdoullahi M, Di Rocco N, Sharifian R (1997). Clinical manifestations in 28 Italian and Iranian patients with severe factor VII deficiency. Haemophilia.

[6] Acharya SS, Coughlin A, Dimichele DM (2004). Rare Bleeding Disorder Registry: deficiencies of factors II, V, VII, X, XIII, fibrinogen and dysfibrinogenemias. J Thromb Haemost.

[7] Mannucci PM, Duga S, Peyvandi F (2004). Recessively inherited coagulation disorders. Blood.

[8] Viswabandya A, Baidya S, Nair SC, Abraham A, George B, Mathews V, Chandy M, Srivastava A (2012). Correlating clinical manifestations with factor levels in rare bleeding disorders: a report from Southern India. Haemophilia.

[9] Peyvandi F, Mannucci PM, Lak M, Abdoullahi M, Zeinali S, Sharifian R (1998). Congenital factor X deficiency: spectrum of bleeding symptoms in 32 Iranian patients. Brit J haematol.

[10] Herrmann FH, Auerswald G, Ruiz-Saez A, Navarrete M, Pollmann H, Lopaciuk S (2006). Factor X deficiency: clinical manifestation of 102 subjects from Europe and Latin America with mutations in the factor 10 gene. Haemophilia.

[11] Mishra P, Naithani R, Dolai T, Bhargava R, Mahapatra M, Dixit A (2008). Intracranial haemorrhage in patients with congenital haemostatic defects. Haemophilia.

[12] Bhattacharya M, Biswas A, Ahmed RPH, Kannan M, Gupta M, Mahapatra M (2005). Clinico-hematologic profile of factor XIII-deficient patients. Clin Appl Thromb Hemost.

[13] Lak M, Peyvandi F, Ali Sharifian A, Karimi K, Mannucci PM (2003). Pattern of symptoms in 93 Iranian patients with severe factor XIII deficiency. J Thromb Haemost.

[14] Peyvandi F, Palla R, Menegatti M, Siboni SM (2012). Coagulation factor activity and clinical bleeding severity in rare bleeding disorders: results from the European Network of Rare Bleeding Disorders. J Thromb Haemost.

[15] Lee CA, Kessler CM, Varon D, Martinowitz U, Heim M (1998). Clinical picture and treatment strategies in factor VII deficiency. Haemophilia.

[16] Siboni SM, Zanon E, Sottilotta G, Consonni D, Castaman G (2012). Central nervous system bleeding in patients with rare bleeding disorders. Haemophilia.

[17] Peyvandi F, Haertel S, Knaub S, Mannucci PM (2006). Incidence of bleeding symptoms in 100 patients with inherited afibrinogenemia or hypofibrinogenemia. J Thromb Haemost.

[18] Peyvandi F, Duga S, Akhavan S, Mannucci PM (2002). Rare coagulation deficiencies. Haemophilia.

[19] Acharya SS, Dimichele DM (2008). Rare inherited disorders of fibrinogen. Haemophilia.

[20] Lak M, Keihani M, Elahi F, Peyvandi F, Mannucci PM (1999). Bleeding and thrombosis in 55 patients with inherited afibrinogenaemia. Brit J haematol.

[21] Peyvandi F, Lak M, Mannucci PM (2002). Factor XI deficiency in Iranians: its clinical manifestations in comparison with those of classic hemophilia. Haematologica.

[22] Peyvandi F, Tuddenham EGD, Akhtari AM, Lak M, Mannucci PM (1998). Bleeding symptoms in 27 Iranian patients with the combined deficiency of factor V and factor VIII. Brit J Haematol.

[23] Shetty S, Madkaikar M, Nair S, Pawar A, Baindur S, Pathare A (2000). Combined factor V and VIII deficiency in Indian population. Haemophilia.

